# 亚肺叶切除术治疗早期非小细胞肺癌的临床研究进展

**DOI:** 10.3779/j.issn.1009-3419.2015.09.07

**Published:** 2015-09-20

**Affiliations:** 200030 上海，上海交通大学附属胸科医院，上海市肺部肿瘤临床医学中心胸外科 Department of Thoracic Surgery, Shanghai Chest Hospital/Shanghai Lung Tumor Clinical Medical Center, Shanghai Jiaotong University, Shanghai 200030, China

1995年肺叶切除术被确立为治疗Ⅰa期非小细胞肺癌（non-small cell lung cancer, NSCLC）的标准术式，而亚肺叶切除术则作为“被迫性术式”予以应用^[1]^。近年来，随着低剂量螺旋计算机断层扫描（computed tomography, CT）在肺癌高危人群筛查中的应用，越来越多的早期肺癌被发现。对于这些早期周围型小病灶肺癌，亚肺叶切除术能否取代肺叶切除术成为标准术式，是目前胸外科领域研究的热点问题^[2, 3]^。

## 1 亚肺叶切除术的手术方式

亚肺叶切除术包括解剖性肺段切除术和楔形切除术。理论上解剖性肺段切除术要优于楔形切除术，主要因为肺段切除术更容易达到合适的切缘距离^[4]^，并且可更彻底地切除段及段间引流的淋巴管，而此淋巴管通常认为是肿瘤复发的重要来源^[5]^。目前研究^[6, 7]^认为肺段切除术要优于楔形切除术，两者相比，解剖性肺段切除术在局部复发率及术后生存率方面要更有优势，特别是对于Ⅰ期NSCLC^[8-12]^（表 1）。El-Sherif等^[10]^回顾性分析了81例经过亚肺叶切除术的病例资料，发现行肺楔形切除术的患者中，只有39%切缘距离超过1 cm，而肺段切除术的患者中有73%超过了1 cm，行楔形切除术的局部复发率要高于肺段切除术。此外Kent等^[4]^也报道了相似的研究结果。不恰当的切缘距离是导致楔形切除术后局部复发率升高的主要原因^[13]^。Schuchert等^[14]^回顾性分析了182例Ⅰ期NSCLC患者的临床资料，所有患者均行解剖性肺段切除术，平均切缘距离是1.82 mm，32例在术后平均14.3个月的时候出现复发转移，其中14例为局部复发，18例为远处转移。在复发转移的患者中，86%的患者切缘距离≤2 cm，并且发现切缘距离与肿瘤直径的比值大于1的患者，其复发率要远低于比值小于1的患者（*P*=0.001, 4）。

**1:** 解剖性肺段切除术与肺楔形切除术的对比研究 Comparison of anatomic segmentectomy and wedge resection

Author	Number of patients	TNM stage	Resection type			5-year survival rate（%）			Local recurrence rate（%）	
			Wedge	Seg		Wedge	Seg		Wedge	Seg
Sugi^[55]^	64	Ia	21	43		95.2	83.7		0	9.5
Sienel^[11]^	87	Ia	31	56		48^*^	71^*^		55	16
Koike^[12]^	328	Ia	112	216		68^#^	91.3^#^		34	6.3
EI-sherif^[10]^	81	I	55	26		-	-		14.5	3.8
Okada^[8]^	158	Ⅰa (≤2 cm)	35	123		85.7	96.7		-	-
Miller^[9]^	25	Ia (≤1 cm)	13	12		27	57		30.8	8.3
-：Not reported；*：5-year cancer-related survival rate；#：5-year disease-specific survival rate.										

## 2 亚肺叶切除术的适应证

目前亚肺叶切除术主要适用于：①高龄、心肺功能差以及患有不能耐受肺叶切除术的合并症；②肿瘤直径≤2 cm的周围型Ⅰa期NSCLC；③肺部CT上表现为磨玻璃密度样（ground-glass opacity, GGO）的结节；④组织病理学类型为原位或微浸润肺腺癌的NSCLC。

### 2.1 高风险患者的治疗

对于高龄、心肺功能差的NSCLC患者来说，行肺部手术的风险相对较高^[15]^。Rostad等^[16]^报道，对 > 70岁的患者行肺叶切除术的死亡率是 < 70岁患者的两倍，主要死于心肺相关的合发症。全肺切除术，特别是右全肺切除术更加不适用于老年患者^[17, 18]^。对于亚肺叶切除术来说，其能有效保留肺功能^[19]^，并且有多项研究^[20, 21]^表明，用于治疗心肺功能差的高龄早期NSCLC患者，与肺叶切除术相比，在手术病死率、术后并发症率、5年生存率及局部复发率方面均无显著统计学差异。

### 2.2 肿瘤大小

肿瘤直径的大小与患者的预后密切相关，目前有研究^[22, 23]^显示肿瘤直径≤2 cm与 > 2 cm的预后显著不同。Carr等^[24]^回顾性分析了429例Ⅰa期NSCLC患者的临床资料，发现肿瘤直径≤2 cm的患者5年无复发生存率为86%，而 < 2 cm的为75%（*P*=0.027）。Nomori等^[23]^分析了179例外周型cT1N0M0 NSCLC患者的临床资料，所有患者均采用解剖性肺段切除加系统性淋巴结清扫术，并且使用术中快速病理来确认切缘距离肿瘤至少2 cm以上，他们发现对于肿瘤直径≤2 cm的患者5年无病生存率（disease free survival, DFS）为96%，而直径在2.1 cm-3.0 cm的患者5年DFS为79%。

### 2.3 GGO病变及特殊病理类型

随着影像学技术的进步，特别是胸部薄层CT（thin-section CT, TSCT）的应用，越来越多的GGO病变被发现。有研究^[25, 26]^显示这些GGO病变通常倾向于原位腺癌（adenocarcinoma *in situ*, AIS）或微浸润腺癌（minimally invasive adenocarcinoma, MIA），对于这些病变来说手术疗效较好^[27]^，因此对这一类NSCLC患者而言，亚肺叶切除术有可能成为其标准术式。2014年，Tsutani等^[28]^分析了239例呈GGO样改变的临床Ⅰa期肺腺癌患者临床资料，其中有90例肺叶切除术、56例肺段切除术、93例肺楔形切除术，术后3年DFS分别为96.4%、96.1%和98.7%，三者无统计学差异（*P*=0.44）。此外，Sugi等^[29]^报道，对于肿瘤直径 < 1.5 cm、GGO成分占比 > 75%的周围型肺癌，行电视辅助胸腔镜手术（video-assisted thoracic surgery, VATS）肺楔型切除的5年DFS可达到100%，而肿瘤直径在1.5 cm-2 cm之间者，行解剖性肺段切除联合纵隔淋巴结采样术的5年DFS为90.5%。

## 3 亚肺叶切除术与肺叶切除术的对比研究

1995年Ginsberg和Rubinstein^[1]^负责的美国肺癌研究组（Lung Cancer Study Group, LCSG）报道了肺叶切除术与亚肺叶切除术治疗Ⅰa期NSCLC的前瞻性多中心随机对照研究结果。该项研究是迄今仅有的一项肺叶与亚肺叶切除术随机对照研究。在该项研究中，122例患者行亚肺叶切除术（包括82例肺段切除术），125例肺叶切除术，结果显示亚肺叶切除组每年的死亡率高达30%，显著高于肺叶切除组，两者之间具有统计学差异。此外，亚肺叶组的局部复发率是肺叶切除组的3倍，而楔形切除术的结果比肺段切除术更差。但该研究显示亚肺叶切除术可有效保留肺功能，在术后12个月-18个月，亚肺叶切除组一秒用力呼气容积（forced expiratory volume in one second, FEV1）下降比例为5.2%，而肺叶切除组为11.1%。此后进一步的研究^[19, 30]^也证实亚肺叶切除能更好保留肺功能。因此作者认为亚肺叶切除术只适用于心肺功能差的患者。自从肺叶切除术成为治疗Ⅰa期NSCLC的标准术式。但随后有学者^[31, 32]^对此研究结果的可靠性提出了质疑，比如亚肺叶切除组楔形切除较多、直径 > 2 cm的肿瘤较多、样本量偏小等，如果肺段切除占比高，结果可能不同。

近年来，随着TSCT在肺部肿瘤筛查中的应用，越来越多的早期肺癌被发现，亚肺叶切除术逐渐受到关注。目前有研究对亚肺叶切除术与肺叶切除术进行了对比（表 2）。研究显示，亚肺叶切除术特别是解剖性肺段切除术与肺叶切除术相比，在术后生存率、局部复发率、术后病死率及术后并发症发生率方面均无明显不同。2014年Landreneau等^[33]^回顾性分析了392例肺段切除术和800例肺叶切除术患者的临床资料，发现两者在5年无复发率（70%, 71%）及5年生存率（54%, 60%）方面无明显不同。Schuchert等^[34]^回顾性分析了107例Ia期肿瘤直径≤1 cm患者的临床资料，发现肺叶切除术、肺段切除术和楔形切除术，三者的复发率和5年生存率无统计学差异。作者随后对785例解剖性肺段切除术患者的回顾性分析^[35]^发现，在术后病理证实为Ⅰa期的NSCLC患者中，肺段切除术和肺叶切除术两者在5年无复发率方面无统计学差异。但有学者并不赞成亚肺叶切除术。Whitson等^[36]^开展的大样本研究显示，在14, 473例Ⅰ期NSCLC患者中接受肺叶切除术的患者，总生存率和癌症特异性生存率更好。Kraev等^[37]^的研究也证实对于肿瘤 < 3 cm的患者，接受肺叶切除术比楔形切除术的生存时间更长。2015年，Zhang等^[38]^发表了一篇迄今为止关于亚肺叶切除术最大样本的*meta*分析，共6, 707例患者进行了亚肺叶切除，15, 219例进行了肺叶切除术，他们发现对于Ⅰ期NSCLC患者行肺叶切除术相关指标要显著好于亚肺叶切除术，并且对于Ⅰa期肿瘤直径≤2 cm的患者来说，肺叶切除术仍然要好于亚肺叶切除术。

**2:** 亚肺叶切除术与肺叶切除术的对比研究 Comparison of sublobar resection and lobectomy

Author	Number of patients	TNM stage	Type of sublobar resection			Mortality (%)			5-year survival rate (%)			Local recurrence rate (%)	
			Wedge	Seg		Lobectomy	Sublobar		Lobectomy	Sublobar		Lobectomy	Sublobar
Tsutani^[28]^	239	Ia	93	56		0	0		97.6	98.7 (Wedge) 98.2 (Seg)		0	0
Kilic^[46]^	184	I	-	78		4.7	1.3		47	46		4	6
Pastorino^[56]^	472	I	61			3	0		49	55		38	36
Sienel^[45]^	199	Ia	-	49		-	-		83	67		5	16
Koike^[39]^	233	Ia (≤2 cm)	14	60		0	0		90.1	89.1		1.3	2.7
Wolf^[48]^	238	≤2 cm	130	24		4	0		80	59		8	16
Campione^[40]^	120	Ia	-	21		3	9.5		65	62		2	19
EI-Sherif^[42]^	784	I	122	85		-	-		54	40		4.2	7.2
Altorki^[50]^	347	Ia	37	16		1	0		86	85		-	-
Billmeier^[47]^	679	I-II	120	35		1.9	7.1		57	49		-	-
Stefani^[57]^	206	≤2 cm	82	-		-	-		85	74		8	22
Chang^[43]^	10761	Ia	2234			-	-		61.4	44		-	-
LCSG^[1]^	247	Ia	40	82		1.6	0.8		69	60		6.4	17
Okada^[51]^	634	Ia	-	155		-	0		94.1☆	95.7☆		3.5	1.9
Iwasaki^[44]^	86	< 2 cm	-	31		0	0		73	70		3.6	3.2
Martin-Ucar^[41]^	34	I	-	17		5.8	5.8		64	70		2	0
Keenan^[30]^	201	I	-	54		4.8	5.6		67^*^	62^*^		7.5	11.1
Kraev^[37]^	289	I	74	-		-	-		5.8#	4.15#		-	-
Variotto^[49]^	411	I	79	14		-	-		64.5	54.5		24.6	39.5
Zhong^[58]^	120	≤2 cm	-	39		0	0		81	79.9		4.9	5.1
-：Not reported；*：4-year survival rate；#：median survival time(years)；☆：3-year survival rate.													

## 4 亚肺叶切除术的随机对照研究

1995年LCSG发表的这项随机对照研究是基于胸片来筛查的肺癌患者，随着胸部CT的普及应用，近年有研究^[52]^报道相对于胸片来筛查肺癌患者，CT减少了肺癌的总体死亡率，并且筛查出的早期肺癌所占比例也越来越大，因此在目前状况下，肺叶切除术是否仍是治疗早期肺癌的首选方式，有不少疑问。为回答这一问题，目前全球范围内正进行二项大型前瞻性多中心随机对照研究。

一项由日本临床肿瘤组（Japan Clinical Oncology Group, JCOG）和西日本肿瘤组（West Japan Oncology Group, WJOG）（JCOG0802/WJOG4607L）联合开展的Ⅲ期多中心随机对照研究^[53]^。该项研究始于2009年8月，全日本71个研究组参与，计划3年入组1, 100例患者。目的是对比肺段切除和肺叶切除治疗周围型NSCLC的生存期。入组标准为：20岁-79岁、心肺功能良好无合并症、周围型肺癌位于肺野的外1/3、单发且肿瘤直径≤2 cm、肿瘤不位于中叶、无淋巴结转移。主要研究终点是总生存期，次要终点是术后肺功能、无复发存活率、局部复发率等。术中要求切缘距肿瘤至少2 cm， < 2 cm必须通过病理确认切缘无肿瘤残留，如果发现切缘阳性或淋巴结转移需转为肺叶切除术。术后随访至少5年，前2年每6个月随访肿瘤标志物和胸片、胸部CT，而后改为每12个月随访1次，计划2015年完成。

另外一项是由美国国家癌症研究所（National Cancer Institute, NCI）联合加拿大149个研究组参与的Ⅲ期多中心随机对照研究（CALGB 140503）^[54]^。目的是对比亚肺叶切除和肺叶切除治疗NSCLC后的DFS。该研究从2007年6月起，计划入组1, 258例患者。入组标准为：肿瘤直径≤2 cm、周围型位于肺野外1/3、术中病理确认无淋巴结转移（右侧4组、7组、10组淋巴结阴性，左侧5组、6组、7组、10组淋巴结阴性）或经术前6周以内的纵隔镜检查无淋巴结转移、体力状态评分0分-2分、5年内无肿瘤史、无放化疗史、年龄≥18岁。主要研究终点是DFS，次要终点是总生存期、局部或全身复发转移率以及术后6个月的肺功能。术后随访前2年每6个月1次，而后改为每年1次，计划2021年完成。

期待这两项研究能对早期NSCLC行亚肺叶切除的临床可行性给予客观评价。

## 5 小结

目前所有支持亚肺叶切除术的研究，仅仅针对Ia期患者，因此术前必须确保分期的准确性，必要时可行PET-CT、纵隔镜或支气管镜超声（endobronchiaultrasound, EBUS）检查以辅助进行纵隔淋巴结分期。此外，相关研究大多是回顾性的，由于术前患者的危险因素，肺癌的组织学类型和大小等各不相同，因此会存在选择性偏倚，更为准确的随机对照研究目前正在进行中，待研究结果公布后，关于亚肺叶切除与肺叶切除的优劣，我们会有一个更加可信的答案。
